# Analysis of biophysical and anthropogenic variables and their relation to the regional spatial variation of aboveground biomass illustrated for North and East Kalimantan, Borneo

**DOI:** 10.1186/s13021-014-0008-z

**Published:** 2014-09-19

**Authors:** Carina Van der Laan, Pita A Verweij, Marcela J Quiñones, André P Faaij

**Affiliations:** 1 Copernicus Institute of Sustainable Development, Group Energy and Resources, Utrecht University, Heidelberglaan 2, Utrecht, 3584 CS The Netherlands; 2 SarVision BV, Agro Business Park 10, Wageningen, 6708 PW The Netherlands

**Keywords:** Aboveground biomass, Tropical forest landscapes, Disturbance, Spatial analysis, Multiple regression, Geographically weighted regression, Biophysical and anthropogenic variables, East Kalimantan, North Kalimantan

## Abstract

**Background:**

Land use and land cover change occurring in tropical forest landscapes contributes substantially to carbon emissions. Better insights into the spatial variation of aboveground biomass is therefore needed. By means of multiple statistical tests, including geographically weighted regression, we analysed the effects of eight variables on the regional spatial variation of aboveground biomass. North and East Kalimantan were selected as the case study region; the third largest carbon emitting Indonesian provinces.

**Results:**

Strong positive relationships were found between aboveground biomass and the tested variables; altitude, slope, land allocation zoning, soil type, and distance to the nearest fire, road, river and city. Furthermore, the results suggest that the regional spatial variation of aboveground biomass can be largely attributed to altitude, distance to nearest fire and land allocation zoning.

**Conclusions:**

Our study showed that in this landscape, aboveground biomass could not be explained by one single variable; the variables were interrelated, with altitude as the dominant variable. Spatial analyses should therefore integrate a variety of biophysical and anthropogenic variables to provide a better understanding of spatial variation in aboveground biomass. Efforts to minimise carbon emissions should incorporate the identified factors, by 1) the maintenance of lands with high AGB or carbon stocks, namely in the identified zones at the higher altitudes; and 2) regeneration or sustainable utilisation of lands with low AGB or carbon stocks, dependent on the regeneration capacity of the vegetation. Low aboveground biomass densities can be found in the lowlands in burned areas, and in non-forest zones and production forests.

**Electronic supplementary material:**

The online version of this article (doi:10.1186/s13021-014-0008-z) contains supplementary material, which is available to authorized users.

## Background

More insights into the spatial variation of aboveground biomass (AGB) are crucial to minimise carbon emissions and global climate change from tropical deforestation, forest degradation and agricultural expansion. According to van der Werf *et al*. globally, approx. 12% of anthropogenic carbon emissions in 2008 were caused by deforestation and forest degradation [1]. During the period 1973–2010, Kalimantan, Indonesian Borneo, has lost ~31% of the total forest area [2]. With regard to land use changes, according to the Governors’ Climate and Forests Task Force Indonesia [3], the recently merged provinces North and East Kalimantan are when combined the third largest carbon emitting provinces in Indonesia, with 255 Mt CO_2_e yr^–1^, after Central Kalimantan (324 Mt CO_2_e yr^–1^) and Riau (258 Mt CO_2_e yr^–1^). According to their ‘business as usual’ scenarios, land use change will cause carbon emissions in North and East Kalimantan to increase to 331 Mt CO_2_e yr^–1^ by 2030 [3]. Mechanisms such as Reducing Emissions from Deforestation and forest Degradation + (REDD+) [4] have been developed to halt such emissions by maintaining lands with high carbon stocks contained in living forest biomass, such as secondary and undisturbed forests. Meanwhile, expansion of low carbon stock agricultural lands can be instead shifted towards areas with already low carbon stocks or AGB, such as abandoned agricultural or restored degraded lands [5] by implementation of sustainable land zoning tools [6].

AGB is not static, but rather spatially and temporally highly variable, particularly in the tropics [7]-[11]. This makes its quantification and the avoidance of high AGB densities or high carbon stocks challenging (it is generally assumed that about half of AGB consists of carbon). As in other tropical forest landscapes, complex matrices of low to high AGB densities can be expected in North and East Kalimantan, due to varying biophysical conditions present, such as terrain and soil types, and anthropogenic disturbances such as fire or logging. For example, forest fires can cause substantial losses in carbon by the emission of large quantities of CO_2_ by the burning of biomass [12],[13], and via logging by the extraction of timber [14],[15]. However, after a fire or logging activities, regeneration can occur, resulting in an increasing sensitivity of the remaining live and dead vegetation to subsequent disturbance events [13],[16]-[19]. Additionally, the type and severity of the disturbance and local biophysical conditions, such as altitude, soil type and the presence of pioneer species, influence the carbon accumulation potential [18],[20]. Therefore, in this paper we address the question of how such biophysical and anthropogenic variables are related to AGB, and contribute to the spatial variation of AGB in a disturbed tropical forest landscape.

AGB can be estimated at forest stand to landscape scale by plot-based measurements [21],[22]. Several existing plot-based studies in tropical forest landscapes have statistically analysed the relationships between AGB and multiple biophysical variables including soil factors [7],[11],[23],[24], altitude [11],[25]-[27] and slope [11],[28]. Anthropogenic variables, however, are usually not considered, while specifically in highly disturbed tropical areas like North and East Kalimantan, these factors are expected to strongly affect AGB. Additionally, anthropogenic variables are important and useful to support the management of, and decision-making on, maintaining carbon stocks in disturbed areas. For these reasons, our analyses include both biophysical and anthropogenic variables.

Other plot-based studies have focused on the impacts of e.g. logging [14],[15] and fire [13],[29], by comparing AGB between undisturbed and disturbed land classes. The relationship between forest cover change and anthropogenic variables has also been analysed [16],[30],[31]. These studies have instead focused on discrete land use and forest classes and therefore have not accounted for local scale AGB variation. Furthermore, the reviewed studies were not spatially explicit or conducted over larger spatial scales, thereby limiting a landscape scale view on the factors that influence the spatial variation in AGB or forest cover.

A variety of spatially explicit data and methods exist to map and monitor land with high and low AGB or carbon over large spatial scales, such as extrapolating plot-based field AGB estimates to vegetation types with remotely-sensed reflectance data and spatial data of biophysical variables [32],[33]. For example, optical data can be used for mapping forest cover, such as Landsat [34]. However, in areas with frequent cloud cover such as the tropics, radar technologies such as ALOS (Advanced Land Observing Satellite) PALSAR (Phase Arrayed L-band SAR) are more suitable [35],[36]. Additionally, the integration of optical and/or radar technologies, including LiDAR (Light Detection And Ranging), has the potential to improve AGB estimates because it may reduce data saturation and mixed pixel problems [35],[37]-[39]. Although the output maps of the aforementioned studies have visualised the spatial distribution of AGB at high resolutions and over large spatial scales, these did not include the effects of biophysical or anthropogenic factors on AGB.

Changes in AGB or carbon stocks have also been modelled at different spatial and temporal scales and resolutions [33],[40]-[43]. Additionally, studies using spatial data for AGB have compared AGB between forest types with different levels of degradation or disturbances, e.g. by logging or fire [36],[44]-[47]. However, the focus was mostly on a single anthropogenic variable, e.g. logging or fire, and interrelationships between or interaction effects amongst variables were not investigated.

The aforementioned studies are useful for the mapping and monitoring of AGB and carbon stocks, for e.g. REDD + mechanisms. To monitor and quantify AGB whilst taking into consideration the high spatial variation, and additionally to enable the modelling of carbon stocks, further analysis of the underlying biophysical and anthropogenic conditions and processes, using a multi-variable approach, is essential. An improved level of information quality, that considered a broader set of variables and their interactions, would allow decision-making to focus on manageable factors in support of land use allocation that minimises carbon emissions and maximises carbon uptake in support of climate change mitigation.

**Figure 1:**
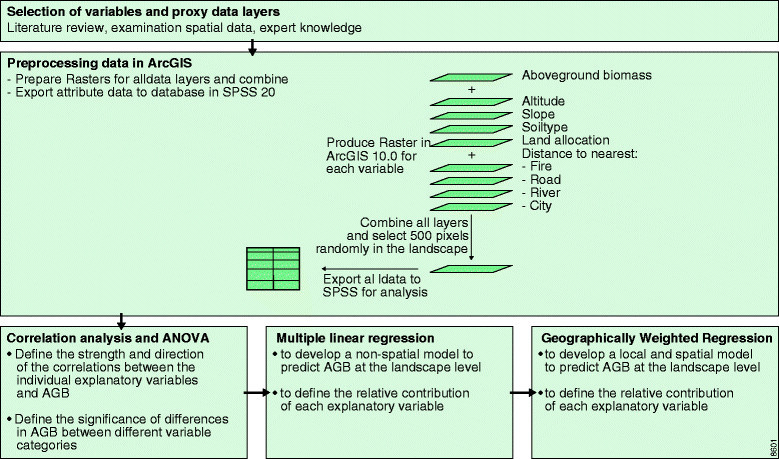
**Schematic overview of the methodological steps in the analysis.**

The aim of this study is to define which of a preselected set of biophysical and anthropogenic variables contribute significantly to the spatial variation of AGB. To this end, statistical analyses were conducted, including analysis of variance (ANOVA), non-spatial multiple linear regression and spatial geographically weighted regression (GWR). An AGB map based on radar remote sensing data and plot-based measurements were utilised, plus landscape scale data on terrain, soil types, land allocation zoning, fires, roads, rivers and cities, covering North and East Kalimantan, Indonesian Borneo (see Figure 1). The results are shown in the Results section and can support the quantification and maintenance of living AGB and carbon stocks. In the Discussion section, the results are discussed in terms of their scientific and societal contribution, followed by the Conclusions and an extensive description of the data and analyses in the Methods section.

## Results

### Relationships between AGB and the continuous explanatory variables

**Figure 2:**
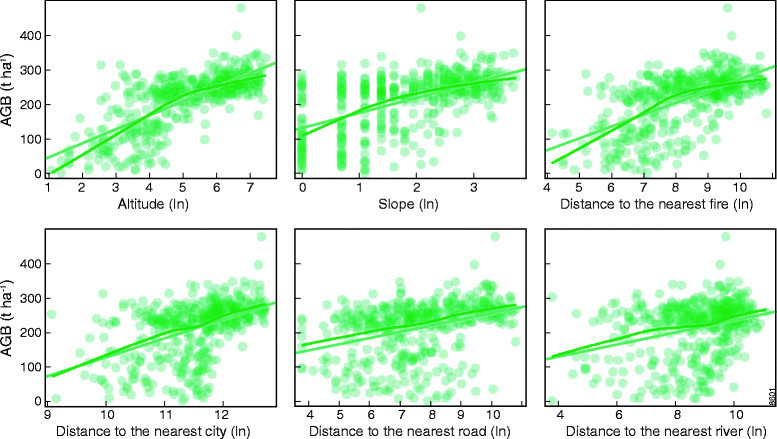
**Relationships between aboveground biomass (AGB, t ha**^**–1**^**) and all continuous variables (logarithmically transformed, ln) in the sample.** The solid circles represent the 465 sample points, the grey line depicts the regression line and the black line depicts the smooth curve.

The distribution of AGB was negatively skewed (Skewness: –0.852, st. error: 0.113, Kurtosis: 0.207, st. error: 0.226), which can be expected in a disturbed tropical forest landscape (Figure S1, in Additional file 1). AGB varied between 2 and 480.0 t ha^−1^ with an overall mean of 213.6 ± 80.1 tha^–1^ (for descriptive statistics, see Table S2, in Additional file 1). AGB and the selected continuous explanatory variables altitude, slope, and distance to the nearest fire, road, river and city (logarithmically transformed) appeared to have a strong, positive correlation (Table S3, in Additional file 1). All relationships are plotted in Figure 2. The Pearson’s correlation coefficients (r) indicated the strongest relationships between AGB and the terrain variables, altitude (r = 0.740, *P* < 0.001) and slope (r = 0.563, *P* < 0.001), and between AGB and distance to the nearest fire (r = 0.607, *P* < 0.001) and city (r = 0.478, *P* < 0.001). Moderately positive relationships were found between AGB and distance to the nearest river and road (r ~ 0.335, *P* < 0.001). Altitude and distance to the nearest city were strongly related to all other explanatory variables (r > 0.400, *P* < 0.001), but not to distance to the nearest river. Distance to the nearest fire was related to the distance to the nearest river and the nearest city. No strong multicollinearity was found (Tolerance >0.200).

### Variation in AGB between altitude ranges and soil types

**Figure 3:**
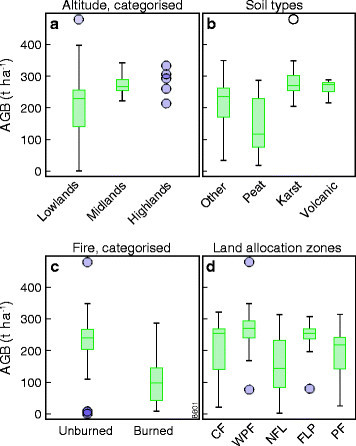
**Boxplots showing the median, the upper and lower quartile for aboveground biomass (AGB, t ha** ^**–1**^ **) in the categorised (a) altitudes; (b) soil types; (c) unburned and burned areas; and (d) land allocation zones (CF, conservation forest; WPF, watershed protection forest; NFL, non-forest land; FLP, forest limited production; PF, production forest).**

The ANOVA on the categorised altitude variable revealed significant differences in mean AGB between the categories lowlands (<750 m), midlands (750–1,500 m) and highlands (>1,500 m), F (2,462) = 32.85, *P* < 0.001. The lowlands (M = 201) had significantly lower AGB than the midlands (M = 276, *P* < 0.001) and the highlands (M = 282, *P* < 0.05). A boxplot of altitude and AGB is shown in Figure 3a. An ANOVA was used to test for mean differences in AGB among four soil types. Means in AGB for soil types differed significantly across the four types (F(3,461) = 14.88, *P* < 0.001). Bonferroni’s post-hoc comparisons on the four soil types indicate that AGB on peatland (M = 142) gave significantly lower means than on karst (M = 261, *P* = 0.001) and volcanic soils (M = 282, *P* < 0.001) (Figure 3b).

### Variation in AGB between burned and non-burned areas and land allocation zones

AGB in non-burned areas (i.e. areas where no fire hotspots were identified by the Moderate-resolution Imaging Spectroradiometer (MODIS) between 2000 and 2008) (M = 223, *P* < 0.001) was significantly higher compared to burned areas (i.e. MODIS fire hotspots were identified within 500 m from the data point between 2000 and 2008) (M = 114, F (1,463) = 79.22, *P* < 0.001). A boxplot of fire and AGB is shown in Figure 3c. Fires were more common in the lowlands (98%) compared to the midlands and highlands. An ANOVA showed significant differences in the mean for AGB between the five land allocation zones (F (4,460) = 56.06, *P* < 0.001) (see also Figure 3d). After Bonferroni’s correction, pairwise comparisons showed that the mean AGB was significantly lower in the non-forest land zone (M = 152, *P* < 0.001) compared to the other categories, and was significantly higher in watershed protection forest (M = 272) and the forest limited production zone (M = 253), compared to production forest (M = 193) and conservation forest (M = 211).

### Multiple linear regression

After removal of the non-significant explanatory variables via conducting a backward multiple linear regression, the variables altitude, distance to the nearest fire, and the categorical variables land allocation zoning and soil type significantly contributed to predicting AGB, and combined explained approx. 59% of the observed variance in AGB in the sample (Adjusted R^2^ = 0.589, F(9,455) = 72.46, *P* < 0.001). The standardised coefficients showed that in this analysis, altitude was the most important explanatory variable (Table 1).

**Table 1:** **Unstandardised coefficients resulting from the non-spatial multiple regression, without (Model 1) and with interaction terms (Model 2)**

Variable	Coefficients Model 1	Coefficients Model 2
Altitude (ln)	32.8 (0.60)**	40.1 (0.73)**
Distance to the nearest fire (ln)	8.1 (0.14)**	8.4 (0.15)**
Soil type		
Karst	43.2*	42.1*
Peat	19.2	25.7
Volcanic	16.8	22.7*
Land allocation zone		
Forest limited production	19.4*	168.8**
Conservation forests	–14.5	–23.1
Production forests	16.5*	41.7
Watershed protection forests	17.1	114.3*
Interaction effects		
Altitude (ln) x Forest limited production		–27.9**
Altitude (ln) x Conservation forests		–1.0
Altitude (ln) x Production forests		–6.6
Altitude (ln) x Watershed protection forests		–18.3*

Standardised coefficients are indicated between brackets; **P <0.001, *P < 0.05; ln, logarithmically transformed.

Altitude and distance to the nearest fire both showed a positive relation with AGB, which means that with increasing altitude and distance to the nearest fire AGB increased. Soil type also made a difference with respect to AGB. Compared to the reference category ‘other’, the categories volcanic and karst showed a higher mean AGB. Karst was the only significant coefficient compared to ‘other’. When compared to the reference category ‘non-forest land’, all land allocation zones showed a higher mean AGB.

In a second model, interaction effects between altitude and land allocation zoning were added (Table 1). These interaction effects added 2% to the explained variance of AGB (R^2^ change = 0.02, F(4, 451) = 5.29, *P* < 0.001). For all land allocation zones, altitude showed a positive effect on AGB; however, this effect was not equally strong in all land allocation zones (Figure S4, in Additional file 1). The strongest relationship between altitude and AGB was found in conservation forests, where altitude explained the AGB variance with about 86% (R^2^ = 0.860, *P* < 0.001). The weakest relationship with altitude was found in watershed protection forests, where altitude explained only 17% of the variance in AGB.

No multicollinearity was present and the standardised residuals showed a normal distribution (Range = [–2.92; 3.96]), meeting the important assumptions of normality and multicollinearity underlying multiple linear regression (see Figure S5, in Additional file 1). However, the Breusch-Pagan test exposed the presence of spatial non-stationarity or heteroscedasticity (Chi-square df = 88.381, *P* ≤ 0.05), invalidating the significance of the statistical tests. The Moran’s I test (Index = 0.147, z-score = 12.02, *P* ≤ 0.05) showed spatial autocorrelation of the standardised residuals, which can cause an unexplained shift in the regression coefficients and can thus influence the output of the model.

### Geographically weighted regression (GWR)

**Figure 4:**
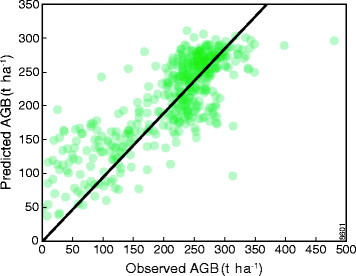
**Relationship between the observed aboveground biomass (AGB, t ha**^**–1**^**) on the AGB map and the predicted AGB generated by the GWR model for the 465 sample points.** The line corresponds to a perfect fit.

Because of the presence of spatial autocorrelation in the standardised residuals of the non-spatial multiple linear regression, geographically weighted regression (GWR) was conducted, producing for each sample point a local relationship between AGB and the explanatory variables. The variables; distance to the nearest road, city and river showed strong multicollinearity with altitude. Finally, three GWR models (Table 2) were computed that did not show multicollinearity; however, Moran’s I test of two of these models showed spatial autocorrelation. In the best model (R^2^ Adjusted = 0.641, *P* ≤ 0.05), the explanatory variables; altitude, distance to the nearest fire, and land allocation zoning were significant, and explained the variation of AGB in the sample with approx. 64% (Table 2). The presence of spatial autocorrelation was unlikely (Index = 0.02, z-score = 1.8, *P* ≤ 0.1). In Figure 4 the AGB values observed on the AGB map are plotted against the AGB values predicted by the model. The standardised residuals showed a normal distribution (Range ~ [–3.80; 4.80]), indicating that the normality assumptions underlying multiple regression were met (see Figure S6, in Additional file 1).

**Table 2:** **Output of the spatial GWR model computed in ArcGIS (** ***P*** < 0.05); * for each variable (ln, logarithmically transformed) the mean of the coefficients is indicated: the GWR produced for each sample point a local model and variable coefficient

	Model
R^2^	0.660
R^2^ Adjusted	0.637
Response variable:	
Observed mean AGB (t ha^–1^)	213.6
Predicted mean AGB (t ha^–1^)	211.9
Explanatory variables:	
Altitude (ln) (mean coefficient)	31.7*
Distance to the nearest fire (ln) (mean coefficient)	8.6*
Land allocation zoning (mean coefficient)	0.17*
Residuals	1.7
Standard Error	46.6
Standardised Residual	0.03

## Discussion

In this study, we combined ANOVA, multiple regression and GWR and used multiple thematic spatial data layers to define which biophysical and anthropogenic variables contributed significantly to the spatial AGB variation in a disturbed tropical forest landscape. Altitude showed the strongest relationship with AGB; individually, and in both regression analyses. This strong positive correlation with altitude is supported for other areas by previous studies e.g. [11],[27]. In our study, the mean AGB was highest in the higher altitudes where volcanic soils are present, and most of the land was allocated to zones where land clearing is not allowed. Moreover, these higher altitudes are less suitable for agriculture and poorly accessible by road. However, by taking into account interaction effects, we found that the influence of altitude on AGB was highest in conservation forest where AGB variation was explained with approx. 86%. It is likely that the forests in these higher, and thus more remote, conservation areas are less impacted by anthropogenic variables, leading to higher AGB densities.

The mean AGB was significantly lower in the lowlands [11],[27]. Lowlands are more susceptible to timber extraction, agricultural expansion and mining because of better accessibility, and typical land allocation zoning where such activities are allowed. Fire is a commonly-used land-clearing method in Indonesia, and has severely affected the study area [16],[48]. Combined with regularly occurring ENSO events, fires that start very locally may quickly spread. In our study, between 2000 and 2008 significantly more fires occurred in the lowlands, and in these burned areas we observed significantly lower AGB [13]. These findings support the results of Fuller *et al*. who found that lowland forests and areas designated for forest conversion in East Kalimantan were more threatened than upland forests and areas not designated for conversion, with slope, elevation, and fire being important factors in determining the threat to forest cover [49].

The contribution of distance to the nearest fire and land allocation zoning is in line with the observations of e.g. Siegert *et al.*[12],[16] and Broich *et al.*[31] that fires, logging, and land clearing contribute substantially to forest cover loss and thus to lower AGB values in North and East Kalimantan. Additionally, Broich *et al.*[31] identified a relatively lower forest cover in 2000 within production forest and non-forest-use zones, and the greatest decrease in forest cover between 2000 and 2008, compared to other land allocation zones. In this study, these differences between the land allocation zones are now also shown for AGB, which may support carbon stock estimations.

Previous studies have focused on the effects of biophysical or anthropogenic factors on forest cover in North and East Kalimantan or Borneo. With the exception of Fuller *et al*. [49], most of these studies, however, included only a limited number of variables, and highlighted the influence of land allocation zoning [31]; land use and fire [30]; or logging and logging roads [2]. In this paper, we provided a reconciliation of these seemingly contradicting results and showed that multiple explanatory variables had a significant effect on AGB and were interrelated to one another. Because of these interrelationships, we underline the importance of including a wide range of biophysical and anthropogenic variables.

Most previous studies have incorporated mainly biophysical variables [e.g. 10, 24–26]. In this study we have demonstrated that in disturbed tropical forest landscapes it is essential to include biophysical and anthropogenic variables in AGB quantification and modelling. Also, the identification of anthropogenic factors that negatively impact AGB can support projections about if and where AGB losses may occur in an area.

We found substantially lower mean AGB on peatland soils (142 t ha^−1^), than was found by Budiharta *et al*. [50] for undisturbed peat swamp forests in Borneo (348.7 t ha^−1^). This may indicate strong anthropogenic disturbances in our study area by for example fires, logging or conversion. Next to aboveground biomass, peatlands also store large amounts of biomass belowground [51],[52]. In regions with large areas of peatland, such as Indonesia [52], the inclusion of belowground biomass in addition to AGB may improve carbon stock predictions.

The GWR showed improved model accuracy (~64%) and a similar ranking of variables compared to the multiple regression; however, soil type did not significantly contribute to this model. This may be caused by an underrepresentation of the limited number of sample points across all soil types.

The variables were selected based on expected relations with AGB, but also by data availability. In order to refine this model for applicability in other landscapes, climate variables such as precipitation and temperature, but also infrastructure data such as logging roads and settlements, may be included, if data is available at an appropriate level of detail.

The root mean squared error between the field-estimated AGB and the AGB from the radar map was 10 t ha^−1^. Although generally this is considered low, it may influence the resulting accuracy of the regression models.

AGB does not only vary spatially, but also temporally because of disturbance and regeneration processes. Temporal analysis of AGB could add a valuable dimension to the present approach, by providing insight into the potential increase or decrease of AGB and carbon stocks.

For maintenance and identification of lands with high AGB, many studies have focused on AGB and carbon mapping and modelling using plot-based and remote sensing data [33]. Many analyses, however, use maps with discrete classes of AGB e.g. [12],[30], and consequently AGB quantification, or modelling, may not be covering the existing high spatial variation sufficiently. Therefore, we recommend using to use AGB maps with a continuous scale.

Methods such as multiple regression and GWR have been utilised for the analysis of, for example; forest attributes [53], the NDVI–rainfall relationship [54], and even for estimating AGB in a tropical forest area [55]. However, using GWR for generating a better understanding of the biophysical and anthropogenic variables that contribute to the regional spatial variation of AGB, by using an extensive spatial dataset, is a relatively new approach. The advantage of using GWR, compared to non-spatial multiple regression, is that it produces a local model, thus accounting for the spatially varying relationships between AGB and the explanatory variables. Moreover, by using GWR the effects of spatial autocorrelation were minimised.

## Conclusions

Better insights into the spatial variation of AGB are needed to support the maintenance of carbon stocks in disturbed tropical forested landscapes. In this paper, we analysed how a set of biophysical and anthropogenic variables were related to, and contributed to the spatial variation of AGB in such a landscape. As was expected for disturbed forest conditions, mean AGB was relatively low and varied strongly throughout the landscape.

Through non-spatial and spatial multiple linear regression, we were able to explain this high spatial variation with, respectively, about 59% and 64%. Because of spatial autocorrelation in the standardised residuals of the non-spatial multiple regression, we conducted GWR. The GWR showed that altitude, distance to the nearest fire and land allocation zoning had the largest significant effect on AGB. Our study showed that mean AGB was relatively higher at the higher altitudes, on karst and volcanic soils, with increasing distance from fire hotspots, in limited production forest, and in watershed protection and conservation forests.

Because of the strong effects of these factors on variation in AGB, efforts to minimise carbon emissions, such as REDD+, should incorporate these factors. This can be implemented through maintenance of lands with high carbon stocks or through the utilisation or regeneration of lands with low carbon stocks or AGB. For example, the maintenance of high carbon stocks should be a priority in the aforementioned zones with high AGB values at higher altitudes. Low AGB or carbon stock lands, such as found in the lowlands in burned areas and in non-forest lands and production forests, should be considered for either regeneration or utilisation purposes, dependent on the regeneration capacity of the vegetation. In these lowland areas, the use of fire should be prevented as much as possible. The utilisation of peatlands should be avoided, especially because of the presence of high belowground carbon stocks. In our study, the variation in AGB was less affected by proximity of roads, rivers and cities.

The high correlations between the explanatory variables showed that the variables were interrelated and thus that AGB variation cannot be explained by one single variable. Instead, spatial analyses should integrate a variety of biophysical and anthropogenic variables to provide a better understanding of spatial variation in AGB.

## Methods

### Study area

The natural resource-rich provinces of North and East Kalimantan (North Kalimantan was established on 25 October 2012 and was previously part of East Kalimantan) have a high spatial variation in biophysical and anthropogenic conditions and processes. For use in this study, the provinces are regarded as one case study region. The terrain consists of undulating slopes and altitudes up to about 2,200 m. Karst and peatlands occur mainly in the lowlands (respectively, ~2% and ~4). The remaining landscape consists mainly of volcanic soils and other soil types (respectively, ~7% and 87%). This landscape is highly dynamic with regard to its past, current and expected land use changes. Until the early 1970s, the original land cover in the lowlands of North and East Kalimantan consisted of extensive dipterocarp forests with high AGB and species richness [18], but driven by forest and land development policies in the 1980s, large-scale degradation, deforestation and conversion to agricultural land have taken place [48]. The main activities were high intensity logging [48], but also large-scale forest fires occurred that were often initiated for land clearing purposes [13],[16],[48],[56], and events associated with El Niño Southern Oscillation (ENSO) [57]. In 1997–98, again very destructive fires related to ENSO occurred, burning 5.2 million ha of North and East Kalimantan’s pristine and logged forests. Hoffmann *et al.*[58] have found that approx. 75% of the burned forests were allocated for logging, timber or oil palm concessions. The frequency and spatial extension of fires have increased over the last few decades in North and East Kalimantan because of deforestation and degradation processes associated with logging, mining and agriculture, and intensifying droughts related to ENSO events [13].

### Selection of variables and proxy data layers

**Figure 5:**
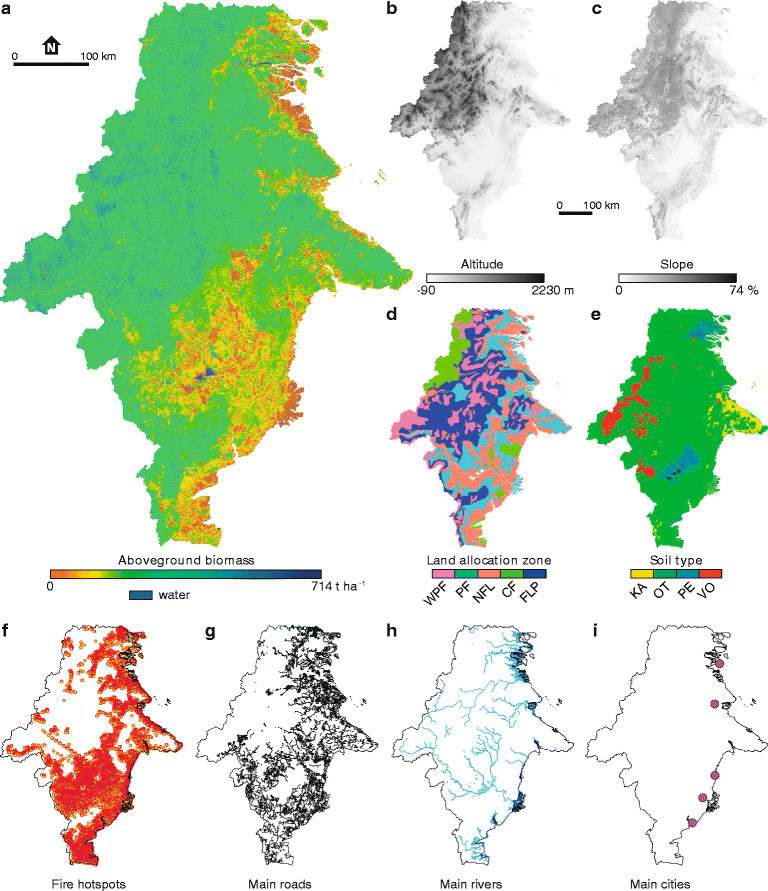
**Data layers for aboveground biomass (AGB) and the biophysical and anthropogenic variables used in this study for North and East Kalimantan.** **(a)** AGB (9 × 9 cells focal means) (t ha^–1^) [38]; **(b)** altitude (m above sea level) [60]; **(c)** slope (%) [60]; **(d)** land (use) allocation zone (WPF, watershed protection forest; PF, production forest; NFL, non-forest land; CF, conservation forest; FLP, forest limited production) (Ministry of Forestry Indonesia, year unknown) [61]; **(e)** soil type (KA, karst; OT, other; PE, peat; VO, volcanic) [63]; **(f)** MODIS hotspots with polygons of 500 m radius [65]; **(g)** main roads [66]; **(h)** main rivers [66]; **(i)** main cities [67].

An overview of the method is given in Figure 1. We included multiple biophysical and anthropogenic variables in the analyses, based on data at a regional scale so that the interrelationships between the explanatory variables could be accounted for. In Figure 5, a landscape-scale view on the data layers is presented in which the landscape-scale pattern for each variable is visible. The use of spatial data enabled the analyses of AGB and several explanatory variables on a continuous scale. The initial selection of the explanatory variables was based on a literature review, field visits, visual examination of spatial data and data availability (for Data sources, see Table S7 and Appendix S8, in Additional file 1).

*The AGB map* (Figure 5a) is based on ALOS PALSAR-LiDAR data and plot-based measurements [38]. Disturbed tropical forest landscapes such as North and East Kalimantan are often covered by clouds or haze. Radar remote sensing is not affected by clouds and has proven to be a remote sensing system responsive to AGB [36],[59]. Saturation of the radar signal at medium AGB levels (150 t ha^–1^) restricts the use of radar remote sensing for a direct radar image inversion into AGB maps. A radar-based forest type map is used in combination with estimated vegetation heights per land cover type, derived from Geoscience Laser Altimeter System (GLAS) LiDAR data, to overcome such saturation effects. This resulted in an AGB map with a resolution of 50 m. Available vegetation height-AGB allometric equations were used to invert heights into AGB values per pixel, overcoming the effect of radar saturation. An accuracy assessment of the AGB map was conducted using field measurements over 54 plots of 0.2 ha over a range of degraded forest types in the study area. AGB values were estimated using the allometric equation developed by Saatchi *et al*. [38],[39]. The accuracy of the AGB map is estimated as 10 t ha^–1^, using the root mean squared error between the field-estimated AGB and the AGB from the radar map for the same location. For more information see Quiñones *et al.*[38].

*Altitude* (Figure 5b) varied from –90 m – 2,230 m in the landscape and was selected because a relationship with AGB is expected [11],[25]-[27]. *Slope* (Figure 5c) was found to have a positive relationship with AGB [8]. Altitude and slope were derived from the Digital Elevation Model (90 m) by the Shuttle Radar Topography Mission (SRTM-DEM) [60]. For the multiple linear regression and GWR, altitude was included as a continuous variable. For the ANOVA, altitude was categorised into several altitude ranges (Lowlands <750 m; Midlands 750–1,500 m; Highlands >1,500 m) (see Appendix S9 in Additional file 1).

*Logging and land conversion* decreases AGB substantially by the harvesting and loss of especially rare tree species [13]. Therefore a relationship is expected between AGB and logging intensity, and thus differences in AGB between protected forested areas, areas allocated for timber or forest concessions, and non-forest land. The data source selected is the land (use) allocation zoning data (Figure 5d) classified by WRI and originally produced by the Ministry of Forestry of Indonesia within the Tata Guna Hutan Kesepakatan (TGHK) mapping program (Ministry of Forestry Indonesia, year unknown) as a proxy for logging and land clearance intensity. The classes designated within this data layer and present in the study area are: ‘forest limited production’, where logging is accompanied by measures to reduce impacts on soil erosion; ‘conservation forest’, which is conservation forest for protected areas; ‘watershed protection forest’, which is intended for watershed protection; ‘non-forest land’ has the status of non-forest use; and ‘production forest’, which is intended for commercial logging [31],[62].

Relationships were reported between AGB and soil drainage [26], soil texture [11], and soil fertility [23],[24]. *Soil type* (Figure 5e) was selected as a proxy, and was included by reclassifying the improved reproduction of the RePPProT land systems map [63] into the categories ‘karst’, ‘peat’, ‘volcanic’, and ‘other’ (for details see Appendices S8 and S9 in Additional file 1).

Forest *fires* (Figure 5f) can occur multiple times at the same spot and in this way can cause substantial losses in AGB [12],[13],[64]. Also the rate of post-fire regeneration depends, amongst others, on the frequency and age of the fire [13],[18]. The proxy for fire included in the multiple linear regression and GWR was ‘distance to the nearest fire’. For the fire data MODIS hotspot data from 2000 to 2008 were used [65], because of its high accuracy recording. According to NASA, each MODIS fire hotspot represents the centre point of a ~ 1 km pixel that contains one or more fires, rather than the exact location of a fire. To overcome this uncertainty, a buffer of 500 m radius surrounding each location was created to define fire hotspot polygons. Additionally, for the ANOVA the fire variable was categorised as burned (≤500 m from a hotspot; i.e. the area within a fire polygon), and non-burned areas (>500 m from a hotspot, i.e. the area outside a fire polygon) (see Appendix S9, in Additional file 1).

Both *roads* (Figure 5g) and *rivers* (Figure 5h) are the primary means of transportation in North and East Kalimantan, and improve accessibility from *cities* (Figure 5i) to forest frontier areas. Therefore, a relationship is expected between AGB and the proxy distance to nearest main road [66], the nearest main river [66] and the nearest main city [67].

### Data pre-processing

In order to reduce the high local-scale variation in AGB caused by natural local variation and by the effect of speckle noise, 9 x 9 cell focal mean statistics was applied to the AGB map in ArcGIS, according to the results generated by Hoekman and Quiñones [68]. All shapefiles were rasterised, and the proximity variables were individually processed by means of the Euclidean Distance tool of the ArcGIS Spatial analyst. For optimal processing, a sample of 500 data points was selected randomly in the data layers with a minimum distance of 1,000 m from one another to minimise the effects spatial autocorrelation ([69]. The data layers were combined and all data queries were exported to a database in SPSS 20. Rows with missing values were deleted, resulting in a dataset of 465 data points.

The continuous explanatory variables showing a skewed distribution were transformed to attain normality. Natural logarithmic (ln) data transformation was in all cases the most suitable of a series of transformations tested for attaining a linear relationship between AGB and the explanatory variables.

### Statistical analyses

Using the Pearson’s correlation coefficient, the strength and direction of the predictive relationship between AGB and each of the continuous explanatory variables were defined. We conducted One-way ANOVA to analyse whether mean AGB among soil types and land allocation zones, among different altitudinal ranges, and between burned and unburned areas was significantly different.

Non-spatial backward multiple linear regression was conducted, and with every step non-significant (p ≥ 0.05) variables were removed one-by-one. The categorical variables land allocation zoning and soil type were included as dummy variables with, respectively, ‘non-forest land’, and ‘other’ as the reference categories. Because land is allocated by the Ministry of Forestry of Indonesia based on climate, slope and soil type, tests for an interaction effect between land allocation zoning and altitude were carried out, by inclusion of product terms in the multiple linear regression [70].

To verify whether the output met the assumptions underlying multiple linear regression, tests for normality and multicollinearity were carried out. To test for normality, we plotted a histogram, a normal PP plot and a normal QQ plot of the standardised residuals. We tested for the presence of significant strong multicollinearity by examining the Tolerance.

Analysing ecological spatial data by multiple linear regression is challenging (e.g. [54],[71], because of the possible existence of spatial autocorrelation and spatial non-stationarity, the latter being the variation in relationships and processes over space. Spatial non-stationarity was tested for by conducting the Breusch-Pagan test on random coefficients. Although often ignored, spatial autocorrelation or the spatial clustering of ecological conditions and processes is a natural, and thus widespread phenomenon [69]. Bini *et al.*[72] indicate that this can cause an unexplained shift in the regression coefficients of global or non-spatial models. To test for the presence of spatial autocorrelation, the Moran’s I Index, the z-score and the p-value for the standardised residuals were calculated. If spatial autocorrelation was present, we additionally conducted GWR in ArcGIS. GWR is a spatial and local form of multiple linear regression that considers and models the spatially varying relationships between explanatory variables and the response variable [53]-[55]. The explanatory variables that showed multicollinearity were excluded from the model.

## Additional file

## Electronic supplementary material

Additional file 1: Figure S1.: Frequency distribution of aboveground biomass in the sample. **Table S2.** Descriptive statistics of the data. **Table S3.** Correlation matrix for the combination of all continuous variables. **Figure S4.** Interaction effects between altitude and land allocation zoning. **Figure S5.** Frequency distribution, PP plot and QQ plot of the standardised residuals of the multiple linear regression. **Figure S6.** Frequency distribution of the standardised residuals of the GWR. **Table S7.** Overview of the variables and the data selected. **Appendix S8.** Data sources. **Appendix S9.** Categorisation of the variables. (PDF 666 KB)

Below are the links to the authors’ original submitted files for images.

Authors’ original file for figure 1

Authors’ original file for figure 2

Authors’ original file for figure 3

Authors’ original file for figure 4

Authors’ original file for figure 5
