# Supplementary material for: Analysis of biophysical and anthropogenic variables and their relation to the regional spatial variation of aboveground biomass illustrated for North and East Kalimantan, Borneo
Source: Carbon Balance Manag. 2014 Sep 19;9:8. doi: 10.1186/s13021-014-0008-z (PMC4168022; doi:10.1186/s13021-014-0008-z)
Supplement: Supplementary file 1 — Additional file 1: Figure S1.: Frequency distribution of aboveground biomass in the sample. Table S2. Descriptive statistics of the data. Table S3. Correlation matrix for the combination of all continuous variables. Figure S4. Interaction effects between altitude and land allocation zoning. Figure S5. Frequency distribution, PP plot and QQ plot of the standardised residuals of the multiple linear regression. Figure S6. Frequency distribution of the standardised residuals of the GWR. Table S7. Overview of the variables and the data selected. Appendix S8. Data sources. Appendix S9. Categorisation of the variables. (PDF 666 KB) [file 13021_2014_8_MOESM1_ESM.pdf]

SUPPORTING INFORMATION

Appendix S1 Frequency distribution of aboveground biomass in the sample

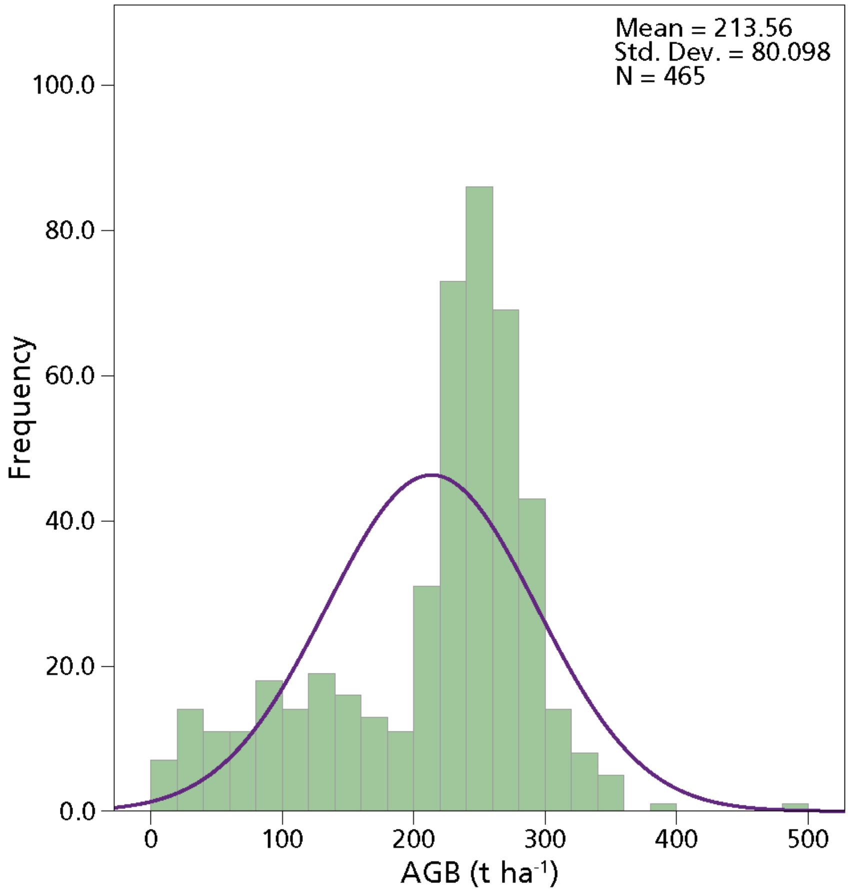

Figure S1. Frequency distribution of aboveground biomass (AGB, t ha^{-1}) (bars) in the randomly selected sample of 465 data points in East and North Kalimantan. The line shows a normal distribution.

Appendix S2 Descriptive statistics of the data

Table S2. Descriptive statistics showing the mean, standard deviation and 95% confidence interval for AGB and the continuous variables in the sample

	Mean	Standard Deviation	95% Confidence Interval	
			Lower	Upper
AGB (t ha^{-1})	213.6	80.1	206.4	220.2
Altitude (m)	367.3	396.5	333.7	401.3
Slope (%)	10.3	8.8	9.5	11.1
Distance to the nearest				
Fire (m)	8248.9	9658.3	7408.3	9061.4
Road (m)	6385.2	9584.2	5579.6	7221.3
River (m)	11352.0	10449.6	10407.7	12381.5
City (m)	127479.2	75899.7	120507.3	134074.6

Appendix S3 Correlation matrix for the combination of all continuous variables

Table S3. Correlation matrix showing Pearson's correlation coefficients (*P* < 0.001) for the combination of all the continuous variables (ln, logarithmically transformed).

	AGB (t ha^{-1})	Altitude (ln)	Slope (ln)	Distance to the nearest			
				Fire (ln)	Road (ln)	River (ln)	City (ln)
AGB (t ha^{-1})	1						
Altitude (ln)	0.740	1					
Slope (ln)	0.563	0.745	1				
Distance to the nearest							
Fire (ln)	0.607	0.696	0.492	1			
Road (ln)	0.369	0.460	0.322	0.439	1		
River (ln)	0.301	0.383	0.182	0.268	0.103	1	
City (ln)	0.478	0.623	0.422	0.390	0.436	0.129	1

Appendix S4 Interaction effects between altitude and land allocation zoning

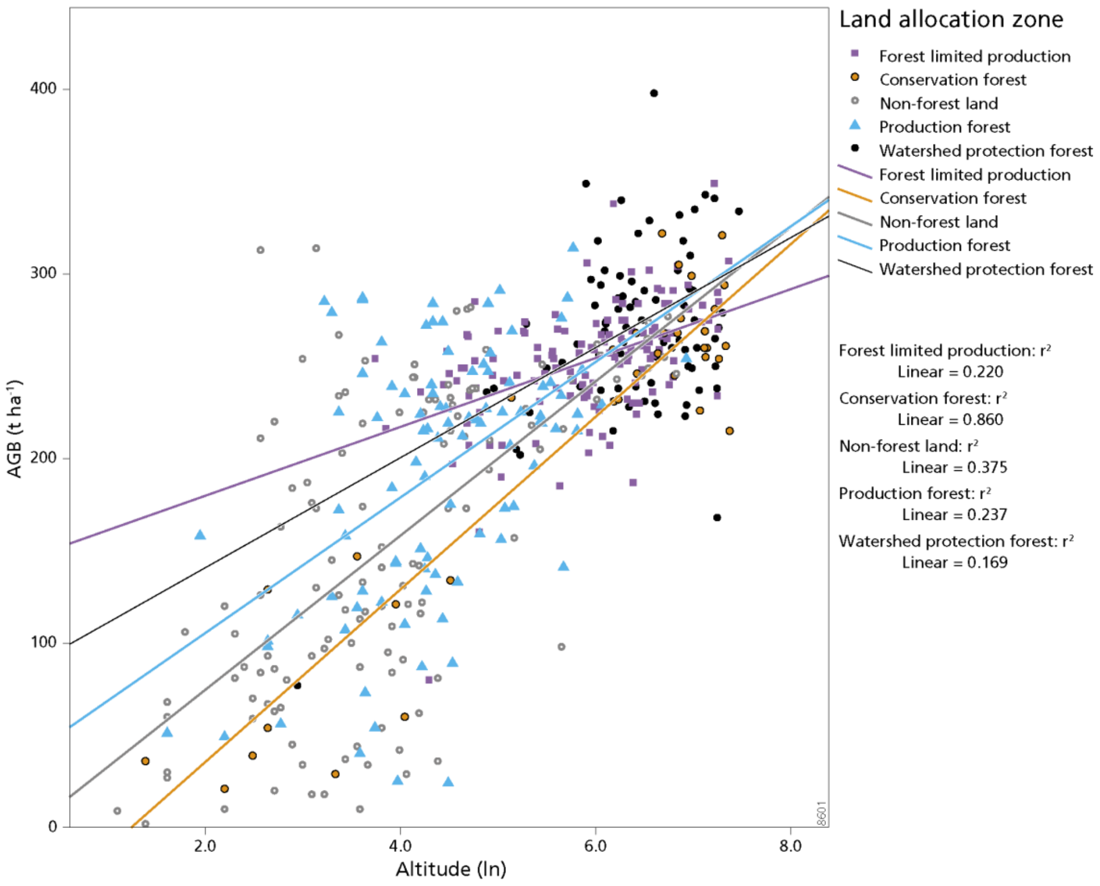

Figure S4: Interaction effect between altitude and the land allocation zones in the multiple regression.

Appendix S5 Frequency distribution, PP plot and QQ plot of the standardised residuals of the multiple linear regression

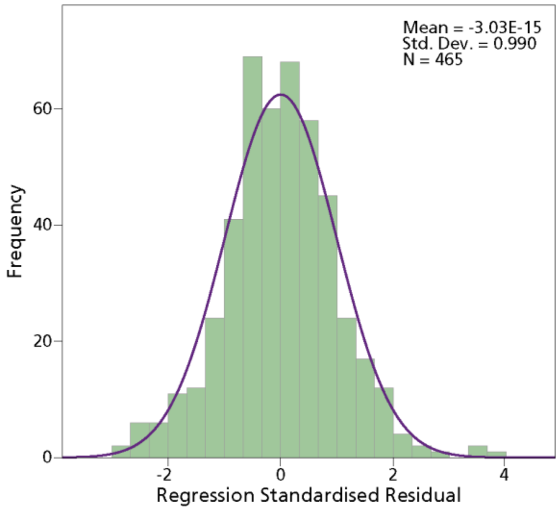

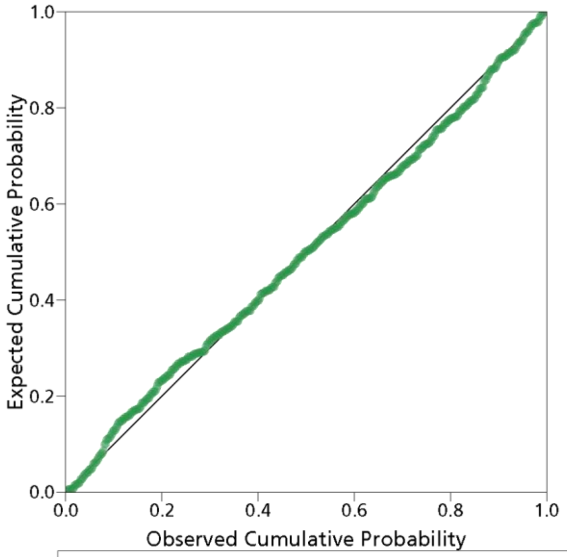

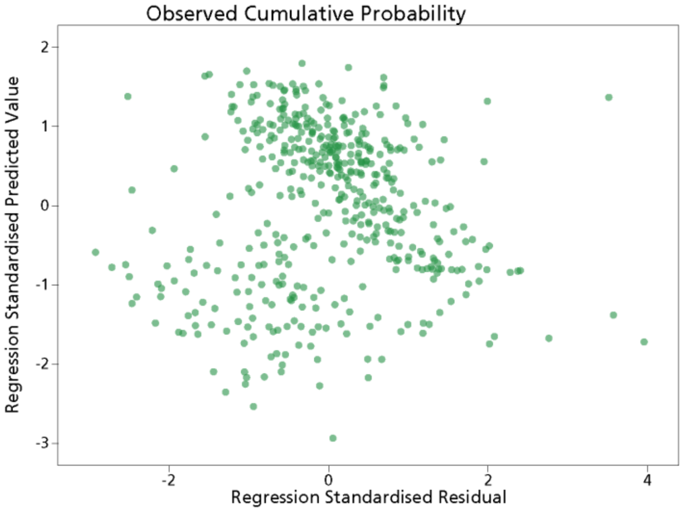

Figure S5 Frequency distribution (a), PP plot (b) and QQ plot (c) of the standardised residuals that resulted from the non-spatial multiple regression model. The lines show a normal distribution.

Appendix S6 Frequency distribution of the standardised residuals of the GWR

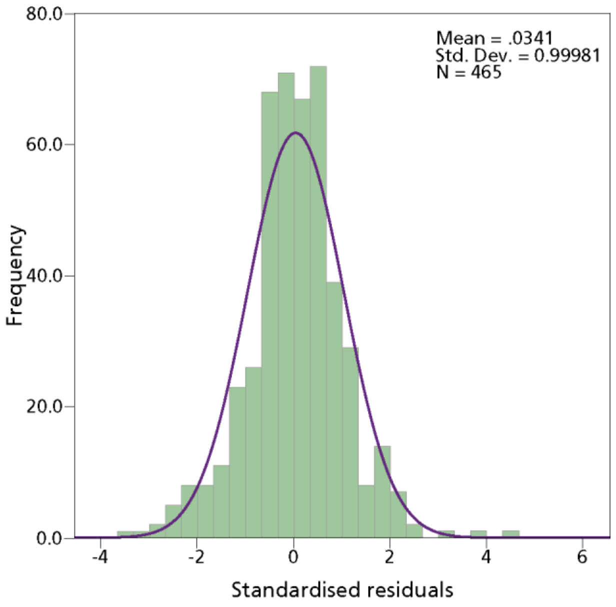

Figure S6. Frequency distribution of the standardised residuals that resulted from the spatial GWR model. The line shows a normal distribution.

Appendix S7 Overview of the variables and the data selected

Table S7. Overview of the variables and the data selected.

Data layer (proxy)	*Year (resolution)*	*Quality data*	*Data and source*
AGB (t ha^{-1})	2008	50m	AGB map (Quiñones *et al.*, 2011)
Altitude (m)	2012	90 m	SRTM-DEM NASA (NASA, 2012)
Slope (%)	2012	90 m	SRTM-DEM NASA (NASA, 2012)
Soil type	1999	Polygon	Geo-corrected and gap filled reproduction of 1:250,000 RePPProT land systems map (Consortium to Revise the HCV Toolkit for Indonesia, 2008)
Land allocation	2009	Polygon	(Ministry of Forestry Indonesia, year unknown)
Distance to the nearest (m)			
Fire	2000-2008	MODIS Point	(NASA/LANCE – FIRMS, 2011)
Road	2003	Polyline	Developed by Bakosurtanal; prepared by WRI for the Interactive Atlas for Indonesia's Forests (Bakosurtanal, 2009)
River	2009	Polyline	Idem (Bakosurtanal, 2009)
City	2012	Point	(CIESIN, 2012)

Appendix S8 Data sources

Aboveground biomass

Quiñones, M.J., Schut, V., Wielaard, N. & Hoekman, D. (2011) Above Ground Biomass map Kalimantan 2008 - Final report. *SarVision Wageningen*, 34p.

Altitude and slope

NASA, 2012. SRTM-DEM (http://www2.jpl.nasa.gov/srtm/)

Soil type

Consortium to Revise the HCV Toolkit for Indonesia (2008) Toolkit for identification of high conservation values in Indonesia. Jakarta, Indonesia. Digital Appendix 12. Ecosystem proxy shapefiles for Kalimantan ver 1.0.

Land allocation

Ministry of Forestry Indonesia (year unknown). Kawasan Hutan (Forest estate) land use maps, General Direktorat of Planning, Ministry of Forestry; downloaded from http://appgis.dephut.go.id/appgis/kml.aspx. Processed and provided by Greenpeace. Prepared by the World Resources Institute (2012). Downloaded from http://www.wri.org/applications/maps/forest-cover-analyzer/

Fire

NASA/LANCE – FIRMS, 2011. MODIS Hotspot / Active Fire Detections. Data set. Acquired on 17-09-2012 online http://earthdata.nasa.gov/data/nrtdata/firms

Main cities

CIESIN, 2012 (Center for International Earth Science Information Network), Columbia University; International Food Policy Research Institute (IPFRI); The World Bank; Centro Internacional de Agricultura Tropical (CIAT): http://sedac.ciesin.columbia.edu/gpw/

Main roads and rivers

Bakosurtanal, 2009. Bakosurtanal , the Indonesian National Coordinating Agency for Surveys and Mapping (http://www.bakosurtanal.go.id). Data available in Minnemeyer et al. (2009). Interactive Atlas of Indonesia's Forests CD-ROM. Washington, DC: World Resources Institute. Prepared by the World Resources Institute (2012).

Appendix S9 Categorisation of the variables

Categorisation of altitude

Lowlands	0 – 750m
Midlands	750 – 1500m
Highlands	> 1500m

Classification of soil type

New class	Original classes (Symbol_LS)
Karst:	GBJ, KPR, OKI
Peat:	BRH, GBT, KLR, MDW
Volcanic:	BTA, BTK, LPN, SMD, TBA
Other:	BKN, BLI, BPD, BRW, GDG, HJA, JLH, KHY, KJP, LHI, LNG, LWW, MGH, MPT, MTL, PDH, PKU, PMG, PST, PTG, RGK, SBG

Categorisation of fire into burned and non-burned areas

burned	≤ 500m hotspot
unburned	> 500m hotspot

References

Bakosurtanal (2009) Bakosurtanal , the Indonesian National Coordinating Agency for Surveys and Mapping (http://www.bakosurtanal.go.id). Data available in Minnemeyer et al. (2009). Interactive Atlas of Indonesia's Forests CD-ROM. Washington, DC: World Resources Institute. Pre.

CIESIN (2012) Center for International Earth Science Information Network, Columbia University; International Food Policy Research Institute (IPFRI); The World Bank; Centro Internacional de Agricultura Tropical (CIAT): http://sedac.ciesin.columbia.edu/gpw/.

Consortium to Revise the HCV Toolkit for Indonesia (2008) Toolkit for identification of high conservation values in Indonesia. Jakarta, Indonesia. Digital Appendix 12. Ecosystem proxy shapefiles for Kalimantan ver 1.0.

Ministry of Forestry Indonesia (year unknown) Kawasan Hutan (Forest estate) land use maps, General Direktorat of Planning, Ministry of Forestry; downloaded from http://appgis.dephut.go.id/appgis/kml.aspx. Processed and provided by Greenpeace. Prepared by the World Resources Institute (2012).

NASA (2012) http://www2.jpl.nasa.gov/srtm/.

NASA/LANCE – FIRMS (2011) MODIS Hotspot / Active Fire Detections. Data set. Acquired on 17-09-2012 online http://earthdata.nasa.gov/data/nrtdata/firms.

Quiñones, M.J., Schut, V., Wielaard, N. & Hoekman, D. (2011) Above Ground Biomass map Kalimantan 2008 - Final report. *SarVision Wageningen*, 34.
