# Supplementary figures and images for: Analysis of biophysical and anthropogenic variables and their relation to the regional spatial variation of aboveground biomass illustrated for North and East Kalimantan, Borneo
Source: Carbon Balance Manag. 2014 Sep 19;9:8. doi: 10.1186/s13021-014-0008-z (PMC4168022; doi:10.1186/s13021-014-0008-z)

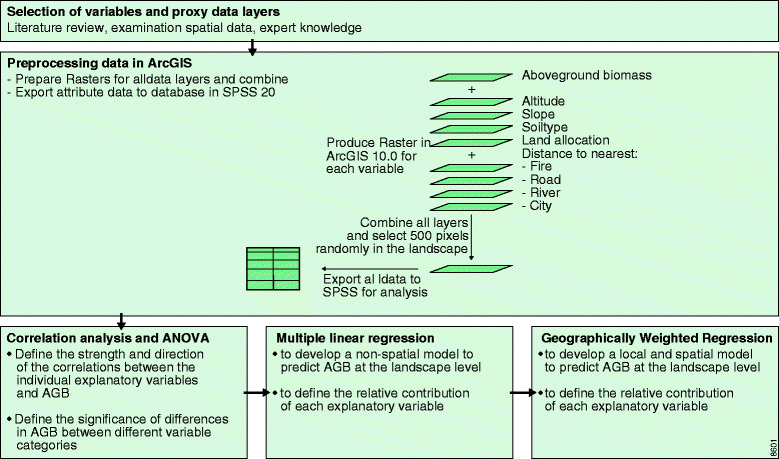

Supplement: Supplementary file 2 — Authors’ original file for figure 1 [file 13021_2014_8_MOESM2_ESM.gif]

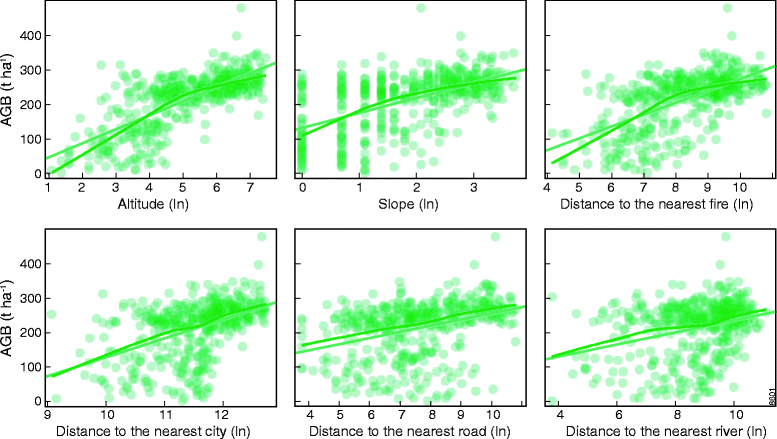

Supplement: Supplementary file 3 — Authors’ original file for figure 2 [file 13021_2014_8_MOESM3_ESM.gif]

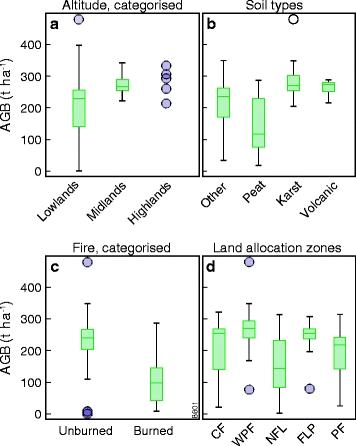

Supplement: Supplementary file 4 — Authors’ original file for figure 3 [file 13021_2014_8_MOESM4_ESM.gif]

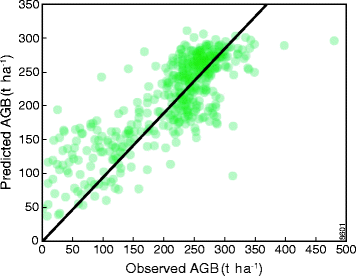

Supplement: Supplementary file 5 — Authors’ original file for figure 4 [file 13021_2014_8_MOESM5_ESM.gif]

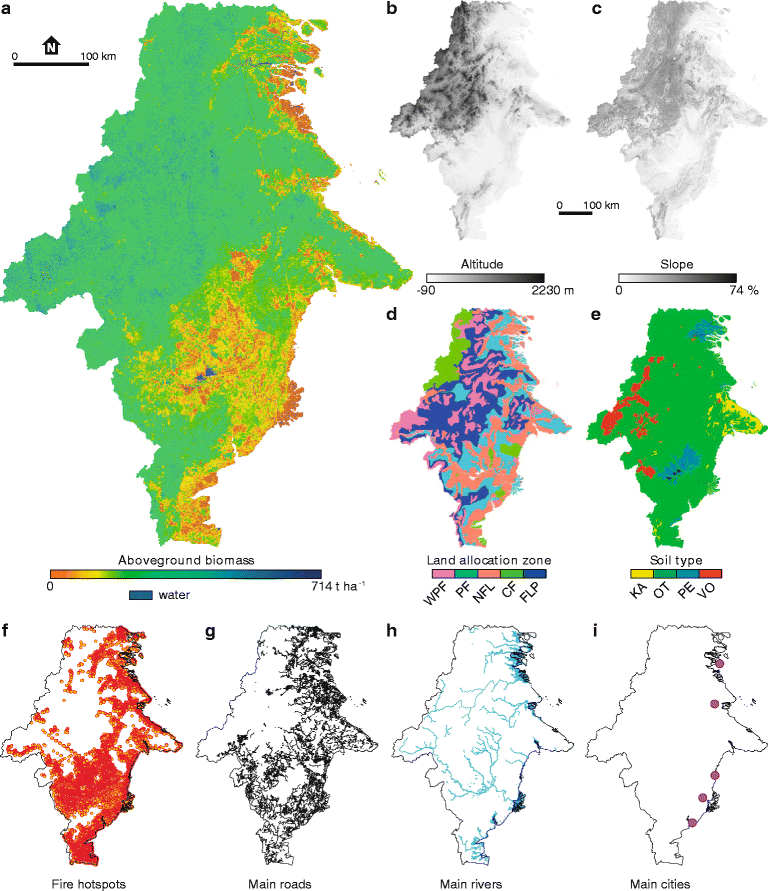

Supplement: Supplementary file 6 — Authors’ original file for figure 5 [file 13021_2014_8_MOESM6_ESM.gif]
